# Learnings from Implementation Strategies to Improve Lipid Management

**DOI:** 10.1007/s11886-024-02174-8

**Published:** 2025-01-08

**Authors:** Nick S. R. Lan, Ruofei Trophy Chen, Girish Dwivedi, Gerald F. Watts, Stephen J. Nicholls, Adam J. Nelson

**Affiliations:** 1 Department of Cardiology, Fiona Stanley Hospital, Perth, WA Australia; 2 Medical School, The University of Western Australia, 35 Stirling Highway, Crawley, Perth, WA 6009 Australia; 3 Harry Perkins Institute of Medical Research, Perth, WA Australia; 4 Victorian Heart Institute, Monash University, Clayton, VIC Australia; 5 Departments of Internal Medicine and Cardiology, Royal Perth Hospital, Perth, WA, Australia; 6 Adelaide Medical School, University of Adelaide, Adelaide, South Australia Australia

**Keywords:** Implementation, Lipid management, Statin, Cardiovascular disease, Adherence

## Abstract

**Purpose of Review:**

Lowering low-density lipoprotein (LDL)-cholesterol reduces cardiovascular risk. International lipid management guidelines recommend LDL-cholesterol goals or thresholds for initiating lipid-lowering therapy. However, contemporary real-world studies have shown that many high- and very high-risk patients are not attaining LDL-cholesterol goals and are not receiving intensive lipid-lowering therapies. In this review, recent examples of implementation strategies for optimising lipid management are discussed.

**Recent Findings:**

Implementation studies are heterogenous in their strategies and design. At the clinician level, multidisciplinary team-based care (including multidisciplinary lipid clinics), pharmacist- or nurse-led interventions, decision-support algorithms or protocols, and educational initiatives have shown potential to improve lipid management. Various strategies to improve patient adherence to lipid-lowering therapies have demonstrated at least short-term efficacy, including education, shared decision-making, behavioural support and nudges. Electronic health records can be leveraged at low cost to identify patients requiring initiation or intensification of lipid-lowering therapies, but the optimal method of integrating automated alerts or nudges to influence decision-making requires further research. Moreover, telehealth and remote care delivery models can improve access to healthcare and facilitate lipid-lowering.

**Summary:**

Multifaceted strategies with a systematic approach to targeting clinician, patient and system related factors can be successful in improving lipid management. Future implementation research should evaluate longer-term outcomes and follow implementation science theories, models and/or frameworks at all stages. By doing so, ongoing implementation studies will help researchers better understand the impact, sustainability and scalability of strategies, and where barriers and facilitators to lipid management may exist in other contexts.

## Introduction

Atherosclerotic cardiovascular disease (ASCVD) remains a leading cause of morbidity and mortality globally despite the increasing availability of therapies that target modifiable risk factors [1]. Epidemiologic, genetic and Mendelian randomisation studies have demonstrated that low-density lipoprotein (LDL)-cholesterol causes ASCVD [2, 3]. Numerous randomised placebo-controlled trials have shown that every 1 mmol/L reduction in LDL-cholesterol level is associated with 21–25% lower risk of ASCVD events [4]. The benefits of reducing LDL-cholesterol levels increase with longer durations of treatment, supporting the concept that cumulative exposure to LDL-cholesterol over time (i.e., “cholesterol-life-years”) leads to atherosclerosis [4–6]. This has led to the modern therapeutic mantra of “the lower the better, the earlier the better” for LDL-cholesterol in the prevention of ASCVD [7]. Current international guidelines therefore recommend LDL-cholesterol goals or thresholds for initiating lipid-lowering therapy that are more intensive than prior guidelines [8, 9]. Yet, there are gaps between guideline recommendations and current clinical practice [10, 11]. Contemporary real-world studies have shown that ~ 80% of high- and very high-risk patients are not attaining guideline-recommended LDL-cholesterol goals [12, 13]. A study of patients with ASCVD found that ~ 40% are prescribed high-intensity statins and < 5% are prescribed ezetimibe or proprotein convertase subtilisin/kexin type 9 (PCSK9) inhibitors, leaving them at preventable increased residual risk [14]. Even if lipid-lowering therapies are prescribed, patients may not adhere or persist with the therapies for multiple reasons, leading to worse outcomes [15–17].

These findings underscore the need to implement strategies to improve lipid management. The EuroPath III project, conducted to optimise LDL-cholesterol management following acute coronary syndrome, established the following five areas of improvement after surveying 555 cardiologists, 445 general practitioners and 662 patients in Europe: (1) inappropriate lipid-lowering therapies prescribed at hospital discharge; (2) lack of guidance to general practitioners regarding lipid-lowering management in the discharge letter; (3) inadequate lipid‐lowering therapy optimisation following discharge; (4) gaps in lipid guideline knowledge and lack of referral practices for general practitioners; and (5) patients’ concerns about lipid management [18]. Given that healthcare delivery is complex, implementation strategies will need to address factors at clinician, patient and system levels. The American and European lipid guidelines were updated in 2018 and 2019, respectively [8, 9]. However, implementing recommendations into practice does not occur without active efforts. Several lipid guidelines now recommend implementation science approaches to enable change in practice [8, 9, 19–22].

In brief, there are 5 major steps in implementation science that can be applied to improving lipid management: (1) identify a gap in evidence-based practice; (2) choose an implementation theory, model or framework; (3) identify barriers and facilitators affecting the translation of evidence into clinical practice; (4) design a strategy to implement the evidence-based practices; and (5) evaluate the success and sustainability of the strategy [21, 23, 24]. This narrative review will focus on recent examples where strategies have been implemented to improve LDL-cholesterol goal attainment, lipid-lowering therapy initiation and/or intensification, and adherence to lipid-lowering therapies in high- or very high-risk patients. We describe papers according to their dominant strategy (clinicians: Table 1, patients: Table 2, system: Table 3), while acknowledging that interventions may target multiple elements. A search of PubMed for articles published within the last 10 years was conducted and supplemented with articles known to the authors.

**Table 1:** Examples of implementation strategies targeting clinician factors

Author	Population	Intervention/Strategy	Main Outcomes
Hess et al*.* (2024) [25]	• 114 hospitalised patients with peripheral arterial disease and LDL-C ≥ 70 mg/dL (1.8 mmol/L) in a single centre in the US	• Randomisation (1:1) to management via a vascular care team including a pharmacist and an algorithm of intensive lipid management or usual care	• Mean LDL-C at 12 months was significantly lower in the intervention group (between-group least-squares mean difference of 43.7%; *p* < 0.0001) • At 12 months, patients in the intervention group were over 3 times and 8 times more likely to achieve LDL-C < 70 (1.8 mmol/L) and < 55 mg/dL (1.4 mmol/L), respectively (*p* < 0.0001)
Jones et al*.* (2021) [26]	• 83 patients seen by a multi-disciplinary lipid clinic in the US during 2019	• A new multi-disciplinary lipid clinic including a cardiologist, genetic counsellor and pharmacist	• 420 patients were referred, and 83 patients were seen (19.8%) • LDL-C reduced by 79 mg/dL (2.0 mmol/L) in patients with FH (*p* < 0.001) • 75% of patients with FH achieved LDL-C < 100 mg/dL (2.6 mmol/L) vs 17% prior to clinic review (*p* < 0.001) • High rates of medication prior authorisation approvals (88% for patients with FH)
Khatib et al*.* (2022) [27]	• First 100 patients seen by a multi-disciplinary lipid clinic in the UK	• A new multi-disciplinary lipid clinic including pharmacists, cardiologists, and lipidologists, that was the sole prescriber of PCSK9 inhibitors in the area	• 48 patients were initiated on a PCSK9 inhibitor (representing > 90% of eligible patients) • Mean LDL-C reduced from 193 mg/dL (5.0 mmol/L) to 81 mg/dL (2.1 mmol/L) (58%; *p* < 0.0001) and was maintained at 12 months
Tucker et al*.* (2024) [28]	• 175 hospitalised patients with peripheral arterial disease who underwent peripheral bypass during admission in a single centre in the US	• 81 patients were managed with a new order set including a preselected consult with a pharmacy lipid protocol and were compared to 94 patients pre-implementation	• More patients were discharged on high-intensity statin compared with pre-implementation (70.4% vs 38.3%; *p* < 0.001) • More patients were discharge with an increased statin intensity compared with pre-implementation (35.8% vs 20.2%; *p* = 0.020)
Hart et al*.* (2024) [29]	• 216 patients with vascular and diabetes-related foot disease from socially deprived areas and treated in a single centre in the UK	• Pharmacist-led virtual lipid clinic implemented within a tertiary referral vascular centre	• LDL-C levels were reduced by 35 mg/dL (0.9 mmol/L) (*p* < 0.001) • The proportion on high intensity statin increased from 39 to 76%) at follow up (*p* < 0.001)
Snaterse et al*.* (2017) [30]	• 754 hospitalised patients with acute coronary syndrome from 11 centres in the Netherlands	• Randomised (1:1) to a nurse-led intervention that included up to 4 clinic reviews in the first 6 months or usual care • Registered nurses had experience in cardiovascular care and were trained in motivational interviewing	• Titration of therapy was more frequent at 6 months in the intervention group compared with usual care (45% vs 24%; *p* < 0.001), which persisted at 12 months (52% vs 34%; *p* < 0.001) • At 6 months, more patients in the intervention attained LDL-C goals (80% vs 69%; *p* < 0.001)
Ruiz-Bustillo et al*.* (2019) [31]	• 78 hospitalised patients with ischaemic heart disease in a single centre in Spain	• Randomised (1:1) to a nurse-led intervention including periodic follow-up, serial lipid levels, and optimisation of therapy or usual care after hospital discharge • The intervention was also incorporated into the cardiac rehabilitation program	• Compared with standard care, patients in the intervention group were significantly more likely to attain LDL-C ≤ 70 mg/dL (1.8 mmol/L) (62% vs 37%; *p* = 0.047) or ≥ 50% reduction in LDL-C (25.6% vs 2.6%; *p* = 0.007) at 6 months • Use of ezetimibe was higher in the intervention group (28% vs 3%; *p* = 0.003)
Omar Khader et al*.* (2024) [32]	• 999 hospitalised patients with acute coronary syndrome from 23 centres in the Netherlands	• A three-step management protocol aiming to reduce LDL-C with steps undertaken every 4–6 weeks: (1) start high-intensity statin; (2) addition of ezetimibe; and (3) addition of PCSK9 inhibitor	• 84% of patients reached LDL-C goal ≤ 70 mg/dL (1.8 mmol/L) with oral medications only • 40 of 102 eligible patients (39.2%) were initiated on PCSK9 inhibitors • After adding PCSK9 inhibitors, 87% achieved their LDL-C goal (*p* < 0.001)
Nakao et al*.* (2022) [33]	• 1,497 hospitalised patients who underwent percutaneous coronary intervention for acute coronary syndrome in a single centre in Japan	• A hospital lipid-lowering protocol that included the prescription of the maximum tolerated dose of statin, ezetimibe, and eicosapentaenoic acid	• Patients treated under the protocol (*n* = 278) were more likely to attain LDL-C ≤ 70 mg/dL (1.8 mmol/L) at 12 months (58% vs 27%; *p* < 0.01) • A greater reduction in LDL-C in the protocol group was seen (− 39.5% vs − 20.4%; *p* < 0.001) compared with pre-intervention • Major adverse cardiovascular events were similar between the groups (16.9% vs 15.5%; *p* = 0.66)
Langer et al*.* (2020) [34]	• 2009 patients with cardiovascular disease or FH across Canada and LDL-C > 77 mg/dL (2.0 mmol/L) despite maximally tolerated statin	• 248 physicians from both primary and specialist care received education about the Canadian lipid guidelines through online reminders which were incorporated into routine practice	• The intervention was adopted by 248 physicians (58% in primary care; 42% in specialist care) • The intervention was associated with a significantly greater proportion of patients attaining LDL-cholesterol < 77 mg/dL (2.0 mmol/L) at visit 2 and 3 (41.7% and 50.8%, respectively; *p* < 0.0001) • PCSK9 inhibitors were prescribed in 27.7% of patients by visit 3

*FH* familial hypercholesterolaemia, *LDL-C* low-density lipoprotein cholesterol, *PCSK9* proprotein convertase subtilisin/kexin type 9, *UK* United Kingdom, *US* United States

**Table 2:** Examples of implementation strategies targeting patient factors

Author	Population	Intervention/Strategy	Main Outcomes
Zafeiropoulos et al*.* (2021) [35]	• 360 hospitalised patients with acute coronary syndrome in a single centre in Greece	• Randomised (1:1) to a physician-led integrated intervention consisting of an educational session at baseline, followed by regular motivational interviewing phone sessions or usual care	• Median PDC was higher in the intervention group than the control group (0.92 vs. 0.86; *p* = 0.03) • The intervention group had increased odds of good adherence (75.5% vs. 66.1%; *p* = 0.04) compared with control • LDL-cholesterol < 70 mg/dL (1.8 mmol/L) was not significantly different between groups at 1 year (49.6% vs 44.9%; *p* = 0.49)
Abughosh et al*.* (2019) [36]	• 152 intervention patients and 304 control patients with suboptimal statin adherence	• Randomised to an initial counselling call and up to 2 monthly follow-up calls by pharmacy students trained in motivational interviewing or usual care	• The mean PDC by statin was significantly higher in the intervention group compared with control (0.67 vs 0.55; *p* < 0.001) • Patients in the intervention group were less likely to discontinue statins (odds ratio 0.38; *p* = 0.006) and were more likely to be adherent (odds ratio 1.87; *p* = 0.008) at 6 months compared with control
Perez de Isla et al*.* (2024) [37]	• 1219 patients across Italy receiving rosuvastatin and ezetimibe as a single-pill combination	• Retrospective analysis of implementing a single-pill combination of rosuvastatin and ezetimibe within the standard care setting	• There was a significantly greater proportion of adherent patients compared with the free-pill combination over a 12-month period (75.2% vs 51.8%; *p* < 0.001)
Chow et al*.* (2022) [38]	• 1,424 patients with acute coronary syndrome from 18 centres in Australia	• Randomised (1:1) to multiple motivational and supportive weekly text messages on medications and healthy lifestyle with the opportunity for 2-way communication (text or telephone) or usual care	• No significant difference in the primary end point of self-reported medication adherence between groups (*p* = 0.15) • No significant difference in LDL-C levels at 12 months between groups (77 mg/dL [2.0 mmol/L] vs 73 mg/dL [1.9 mmol/L]; *p* = 0.34)
Horne et al*.* (2022) [39]	• 182 patients taking with coronary artery disease and an indication for statin from cardiology clinics in the US	• Randomised (1:1) to nudges or control; nudge content, timing, frequency, and delivery route (text messages, emails, voice calls, etc.), were personalized by artificial intelligence	• After 12 months, the PDC by statin therapy was significantly greater in the nudge group compared with control (0.74 vs 0.64; *p* = 0.04) • Adherent subjects (PDC ≥ 80%) were more concentrated in the nudge group (66.3% vs 50.5%; *p* = 0.04)
Reddy et al*.* (2022) [40]	• 162 patients with known coronary artery disease and poor adherence in the US	• Randomised to: (1) control group that received a pill-monitoring device with no alarms or feedback; (2) an individual feedback group that received a daily alarm and a weekly medication adherence feedback report; or (3) a partner feedback group that received an alarm and a weekly feedback report that was shared with a friend, family member or a peer	• During the 3-month intervention, statin adherence was higher in both feedback arms (individual 89% and partner 86% versus control 67%; *p* < 0.001 and *p* = 0.001, respectively) • At 6 months, there was no difference in statin adherence between either of the feedback groups and the control (individual 60% and partner 52% versus control 54%; *p* = 0.75 and 0.97, respectively)

*LDL-C* low-density lipoprotein cholesterol, *PDC* proportion of days covered, *US* United States

**Table 3:** Examples of implementation strategies targeting health system factors

Author	Population	Intervention/Strategy	Main Outcomes
Adusumalli et al*.* (2023) [41]	• 158 clinicians from 28 primary care practices including 4,131 patients who met criteria for statin therapy in the US	• Cluster randomised to clinician nudge (active choice prompt in the EHR during the patient visit and monthly feedback on prescribing patterns compared with peers), patient nudge (an interactive text message delivered 4 days before the visit), combined nudge or usual care	• After 6 months, the clinician nudge increased statin prescribing by 5.5 percentage points (*p* = 0.01) • The combined clinician and patient nudge increased statin prescribing by 7.2 percentage points (*p* = 0.001) • The patient nudge alone did not affect statin prescribing (0.9 percentage points; *p* = 0.32)
Adusumalli et al*.* (2021) [42]	• 82 cardiologists and 11,693 patients eligible for statin therapy from 16 cardiology practices in the US	• Cluster randomised to passive choice (clinicians had to manually access an alert embedded in the EHR to select options to initiate or increase statin therapy), active choice (an interruptive EHR alert that prompted the clinician to accept or decline guideline-directed statin therapy) or usual care	• In adjusted analyses, the change in statin prescribing rates at optimal dose over time was not significantly different from control for passive choice (*p* = 0.86) or active choice (*p* = 0.08) • In the subgroup of patients with cardiovascular disease, statin prescribing at the optimal dose increased by 3.8 percentage points in the active choice group (*p* = 0.008)
Shah et al*.* (2024) [43]	• 96 cardiovascular and internal medicine clinicians in the US and 2,500 patients	• Cluster randomised to receive EHR alerts with recommendations or no alert for outpatients with very high-risk atherosclerotic cardiovascular disease and LDL-C ≥ 70 mg/dL (1.8 mmol/L)	• The proportion of patients where therapy was intensified at 90 days was numerically higher in the alert group (14.1% vs 10.1%; *p* = 0.08) • Intensification was twofold greater among patients whose clinicians did not dismiss the alerts (21.2% vs 10.4%; *p* < 0.001)
Asch et al*.* (2015) [44]	• 340 primary care physicians and 1,503 patients at elevated cardiovascular risk in 3 primary care practices in the US	• Physicians were randomly assigned to control, physician incentives ($1,024 per enrolled patient meeting LDL-C goal), patient incentives ($1,024 distributed through daily lotteries tied to medication adherence), or shared physician–patient incentives	• At 12 months, only patients in the shared physician–patient incentives group achieved reductions in LDL-C levels statistically different from those in the control group (8.5 mg/dL or 0.2 mmol/L; *p* = 0.002) • 49% of the shared group attained LDL-C goals, compared to 40% in both the physician and patient incentive groups and 36% in the control group (*p* = 0.03)
Barankay et al*.* (2020) [45]	• 805 patients at elevated cardiovascular risk, suboptimal LDL-C control and evidence of imperfect adherence to statin across multiple healthcare settings in the US	• Randomised to control or 3 intervention groups: (1) The sweepstakes group involved incentives for daily adherence; (2) in the deadline sweepstakes group, incentives were reduced if participants were adherent only after a reminder; and (3) the sweepstakes plus deposit contract group split incentives between daily adherence and a monthly deposit reduced for each day of nonadherence	• After 6 months, adherence was greater in those receiving financial incentives (*p* < 0.001 for each incentive group vs control) • However, the change in LDL-C from baseline to 12 months was not significantly different compared with the control group (adjusted *p* > 0.99 for each incentive group vs control)
García et al*.* (2022) [46]	• 346 patients hospitalised for acute coronary syndrome in a single centre in Spain	• A virtual lipid clinic providing monthly telephone follow-up calls and involving a multidisciplinary team of cardiologists, residents and a nurse; treatments were adjusted according to a therapeutic algorithm	• Definitive therapy consisted of high-intensity statins (32.4%), high-intensity statins plus ezetimibe (56.9%) and PCSK9 inhibitors (10.7%) • At 12 months, LDL-C levels were reduced from 108.4 mg/dL (2.8 mmol/L) to 48.7 mg/dL (1.3 mmol/L) • 95.4% and 81.5% of patients attained LDL-C < 70 mg/dL (1.8 mmol/L) and < 55 mg/dL (1.4 mmol/l), respectively
Blood et al*.* (2023) [47]	• 10,803 patients with blood pressure and/or LDL-C above guideline-recommended goals, from a diverse healthcare network in the US	• Enrolled patients received education, home blood pressure device integration, and medication titration but 1,266 patients requested education only without medication titration • Non-licensed navigators and pharmacists, supported by clinicians, coordinated care using standardised algorithms, task management and automation software, and omnichannel communication	• At 12 months, participants in the medication management cohort observed significant reductions in LDL-C (mean reduction of 37.5 mg/dL or 1.0 mmol/L), compared with minimal changes (mean reduction 10.2 mg/dL or 0.3 mmol/L) in the education-only cohort • Similar rates of LDL-C reductions were observed across different racial, ethnic, and primary language groups
Nissen et al*.* (2022) [48]	• 1,196 participants who responded to advertisements, completed the Web application assessment and signed informed consent	• A technology assisted self-selection Web application to qualify participants without a medical background for a non-prescription moderate-intensity statin based on current guidelines	• The web application self-selection resulted in an outcome concordant with clinician assessment in 90.7% and 98.1% had a concordant final use outcome during treatment • Mean percent change in LDL-C was 35.5% in the study cohort
Ruiz-Bustillo et al*.* (2022) [49]	• 462 hospitalised patients with acute coronary syndrome in a single centre in Spain	• A mobile device-based intervention including periodic virtual follow-up, serial LDL-C (first test at 6 weeks and then every 6 weeks after therapy changes), and optimisation of therapy using a pre-specified algorithm and electronic prescriptions	• At the end of the 3-month optimisation period, 327 (70.7%) patients had LDL-C ≤ 70 mg/dL (1.8 mmol/L) • Overall, 38.5% of patients had changes in lipid-lowering therapy • At 1 year, 71.1% of those with LDL-C measured (68.6% of cohort) attained LDL-C ≤ 70 mg/dL (1.8 mmol/L)

*EHR* electronic health record, *LDL-C* low-density lipoprotein cholesterol, *PCSK9* proprotein convertase subtilisin/kexin type 9, *US* United States

## Clinician-level Strategies

### Multidisciplinary Team-based Care

Team-based collaborative care is a key strategy to address therapeutic inertia [8, 9]. An example of a recent implementation study is the OPTIMIZE PAD-1 trial, which was a pragmatic randomised trial conduced in a single centre in the United States [25]. In the trial, 114 patients with peripheral arterial disease and LDL-cholesterol ≥ 70 mg/dL (1.8 mmol/L) were randomised to multidisciplinary vascular care team management that included pharmacist reviews and an intensive lipid management algorithm or usual care supplemented with healthcare provider education [25]. At 12 months, patients in the vascular care team group had a significantly lower LDL-cholesterol (between-group least-squares mean difference of 43.7%; *p* < 0.0001) and were 8 times more likely to attain an LDL-cholesterol < 55 mg/dL (1.4 mmol/L; *p* < 0.0001) compared with patients in the usual care group [25]. Use of combination lipid-lowering therapy (including PCSK9 inhibitors) by 6 months was significantly greater in patients treated by the vascular care team compared with usual care (61.4% vs 5.3%; *p* < 0.0001) [25]. The study also demonstrates that the underutilisation of therapies may not simply reflect knowledge gaps alone among healthcare providers, as education did not significantly alter care in the usual care group [25]. Team-based care helps to overcome some of the barriers that healthcare providers may face such as adequate time, resources and support when rapidly intensifying lipid-lowering therapies in a stepwise approach.

The effectiveness of team-based care is also exemplified by multidisciplinary lipid clinics. In an implementation study, the first year of a new multidisciplinary lipid clinic in the United States was evaluated using the RE-AIM framework and a pre/post-study design [26]. Although the investigators found limited reach and adoption of the clinic, there was an increased prescribing of evidence-based therapies, high rates of medication prior authorisation approvals, and significant reductions in lipid levels in patients with severe lipid conditions [26]. In patients with familial hypercholesterolaemia for example, 75% attained LDL-cholesterol levels < 100 mg/dL (2.6 mmol/L) after clinic review compared with 17% prior to clinic review [26]. Similarly, a multidisciplinary lipid clinic designed to address the poor uptake of PCSK9 inhibitors assessed and followed up patients potentially eligible for PCSK9 inhibitors in the United Kingdom [27]. The clinic initiated PCSK9 inhibitor therapy in > 90% of eligible patients, maintained a 65% reduction in LDL-cholesterol levels at 12 months, was considered cost-effective and was well-received by patients [27]. However, the resources available for team-based management may differ between health systems, limiting their generalisability.

### Pharmacist- or Nurse-led Interventions

Pharmacist-led interventions have the potential to improve medication adherence and depending on their complexity, increase the prescribing of, and access to, lipid-lowering therapies [50–52]. The common types of pharmacist-led interventions for lipid management include prompting of physicians or patient contact [52, 53]. A recent single-centre study with a pre/post-study design in 175 hospitalised patients with peripheral arterial disease in the United States demonstrated that the addition of a pharmacist-led lipid consult and protocol was significantly associated with more patients being discharged on high-intensity statin (70.4% vs 38.3%; *p* < 0.001) or an increased statin intensity (35.8% vs 20.2%; *p* = 0.020) [28]. Also, another single-centre study of 216 vascular and diabetic foot patients in an area of high social deprivation in the United Kingdom demonstrated that a pharmacist-led lipid clinic significantly improved the utilization of high-intensity statins (76% vs 39%; *p* < 0.001) and other therapies, with more patients attaining lipid goals [29]. A systematic review and meta-analysis of 26 randomised trials (22,095 patients) found that pharmacist-led interventions significantly reduced LDL-cholesterol levels compared with usual care, with greater observed benefits in studies where LDL-cholesterol was the sole primary outcome [52]. A high degree of heterogeneity between studies was noted and the optimal pharmacist-led intervention to improve lipid management over the long term requires more research [52].

Nurse-led interventions have shown promise in improving several aspects of primary and secondary prevention of ASCVD [54]. Like dedicated pharmacist-led interventions, nurse led-interventions can permit time and resources to conduct patient education on medication intensification, adherence and side effects. For example, in a multicentre implementation study of 754 patients with acute coronary syndrome in Netherlands, patients were randomised to a nurse-led intervention that included up to 4 clinic reviews in the first 6 months after hospital discharge or usual care [30]. At 6 months, titration of lipid-lowering therapy was more frequent in the intervention group (45% vs 24%; *p* < 0.001) compared with usual care, and this persisted at 12 months follow-up (52% vs 34%; *p* < 0.001) [30]. Furthermore, another recent single-centre randomised trial in Spain evaluated the impact of a nurse-led intervention that included periodic follow-up to optimise lipid-lowering therapy after hospital discharge in patients with an ischemic heart disease event [31]. Compared with standard care, patients in the intervention group were significantly more likely to attain LDL-cholesterol ≤ 70 mg/dL (1.8 mmol/L) (62% vs 37%; *p* = 0.047) or ≥ 50% reduction in LDL-cholesterol (25.6% vs 2.6%; *p* = 0.007) at 6 months [31]. The use of ezetimibe was significantly higher in the intervention group (28% vs 3%; *p* = 0.003) [31]. Ongoing studies should assess the impact of similar implementation strategies on the prescribing of PCSK9-directed therapies.

### Protocols or Algorithms

The implementation of protocols to identify patients at risk and optimise lipid management may help overcome barriers relating to lack of guideline adherence, lack of local standardised procedures and insufficient LDL-cholesterol monitoring [55]. A review has highlighted some of the initiatives that have improved in-hospital and post-discharge lipid management in patients with acute coronary syndrome in Europe [55]. The PENELOPE study for example, is a multicentre, prospective, non-randomised trial in the Netherlands which showed that stepwise lipid-lowering management protocols can be feasibly implemented to improve outcomes [32]. Overall, 87% of 999 very high-risk patients attained LDL-cholesterol ≤ 70 mg/dL (1.8 mmol/L) and 40 of 102 eligible patients (39.2%) were initiated on PCSK9 inhibitors [32]. In another single-centre observational study with pre/post study design in Japan, the implementation of an in-hospital lipid-lowering management protocol was associated with significantly more patients with acute coronary syndrome attaining LDL-cholesterol ≤ 70 mg/dL (1.8 mmol/L) at 12 months (58% vs 27%; *p* < 0.01) [33]. However, we have found that implementing algorithms of care alone will not lead to sufficient changes in lipid management, even in very high-risk patients [56, 57]. The uptake of new protocols, akin to guidelines, is at the discretion of clinicians [56, 57]. Indeed, adherence to the lipid-management protocol was only 29% in the Japanese study [33]. Ongoing education may be required to encourage adherence and prevent relapse, especially in busy hospital settings where staff changeover is frequent and competing priorities are common [58].

### Clinician Education

Suboptimal clinician awareness of guideline recommendations represents an important barrier to lipid management [59, 60]. LDL-cholesterol goal attainment may be more common among patients whose clinicians’ knowledge of lipid goals is consistent with guidelines [61]. However, there are few recent studies assessing the impact of clinician education on lipid management. GOAL Canada is an example of an implementation study where 248 physicians across Canada received education about the Canadian lipid guidelines through online reminders after their consultation with a patient with ASCVD not attaining LDL-cholesterol goal [34]. The intervention was associated with a significantly greater proportion of patients attaining LDL-cholesterol < 77 mg/dL (2.0 mmol/L) at visit 2 and 3 (41.7% and 50.8%, respectively; *p* < 0.0001) [34]. In addition, PCSK9 inhibitors were prescribed in 27.7% of patients by visit 3 [34]. Interestingly, the 4 top reasons for not prescribing PCSK9 inhibitors were cost (26%), “not needed” (27%), patient refusal (25%) and “will prescribe next visit” (18%) [34]. Thus, gaps exist in both knowledge (e.g., risk underestimation) and action (not intensifying treatments) [34]. This further highlights the issue of clinical inertia, where clinicians do not initiate or intensity lipid-lowering therapies despite being aware that their patient’s LDL-cholesterol is not at goal [62]. Here, GOAL Canada demonstrates the value of clinician-oriented education in addressing knowledge and care gaps. Using existing electronic health records as a platform makes GOAL Canada potentially cost-effective and generalisable [34].

Whilst implementation of guidelines requires clinician knowledge, the prescribing of lipid-lowering therapy can be influenced by beliefs regarding their benefits and risks [63]. The PALM registry showed that patients treated by clinicians with concerns regarding statin safety were less likely to attain LDL-cholesterol < 100 mg/dL (2.6 mmol/L), highlighting that knowledge of the guidelines is not the only limiting factor [63]. Moreover, many primary care physicians feel that lipid management in patients with acute coronary syndrome is the responsibility of specialists and are not familiar with prescribing PCSK9 inhibitors [60]. Future interventions should target clinician beliefs whilst providing education on non-statin therapies. An ongoing multicentre study, LOGAN-CV, will evaluate the impact of a multifaceted intervention to improve clinician adherence to guidelines and the proportion of patients attaining LDL-cholesterol goals [64]. LOGAN-CV will provide clinicians with educational modules, newsletters, a cloud-based platform summarising LDL-cholesterol management performance, peer-to-peer calls, and pre/post surveys [64].

## Patient-level Strategies

### Patient Education

Adherence to medications is a complex and dynamic issue [65–67]. A limited appreciation and inadequate knowledge of the benefits of reducing LDL-cholesterol represents a barrier to adherence [66]. In addition, patients may refuse or discontinue lipid-lowering therapies due to a fear of actual or perceived side effects [68]. Negative news stories about statins have resulted in misperceptions and an increased rate of discontinuation [69, 70]. Indeed, data from the PALM registry found that over 1 in 4 adults believe that statins cause memory loss [68]. Statin-associated muscle symptoms impede adherence and intensification, with rates in real-world studies that are higher than expected (likely due to nocebo effect) compared with clinical trials [71–73]. Of note, bempedoic acid has been shown to reduce ASCVD events in patients with statin intolerance and represents an alternate option, but is not available in many countries [74]. Nonetheless, patient-centred education to increase patient understanding, and shared decision-making to align patient and clinician goals, remains crucial to improving adherence [75, 76]. An example of implementing patient-oriented education is seen in the IDEAL-LDL trial, which was a single-centre pragmatic randomised trial conduced in Greece [35]. The trial randomised 360 hospitalised patients with acute coronary syndrome to physician-led education (including a 15–20 min in-person discussion) followed by motivational interviewing by phone call at 1 and 6 months or usual care [35]. The median proportion of days covered by lipid-lowering therapy was significantly higher in the intervention group (0.92 vs 0.86; *p* = 0.03) compared with usual care, but attainment of LDL-cholesterol < 70 mg/dL (1.8 mmol/L) was not significantly different between groups at 1 year (49.6% vs 44.9%; *p* = 0.49) [35]. Good adherence however, was associated with LDL-cholesterol goal attainment in both groups [35]. The study provides further insights into practical implementation of patient education as part of routine care [35].

### Behavioural Support

Motivational interviewing is a patient-centred form of counselling that can improve adherence in patients with chronic conditions through promoting behavioural changes and strengthening motivation [77]. Extending this strategy to phone-based delivery of motivational interviewing, a randomised trial was recently conducted in patients in the United States who were nonadherent to statins [36]. Patients were randomised to an initial counselling call plus up to 2 monthly follow-up calls (*n* = 155) or to control (*n* = 304) [36]. The mean proportion of days covered by statin was significantly higher in the intervention group (0.67 vs 0.55; *p* < 0.001) compared with control [36]. Patients in the intervention group were less likely to discontinue statins (odds ratio 0.38; *p* = 0.006) and were more likely to be adherent (odds ratio 1.87; *p* = 0.008) at 6 months compared with control [36]. However, many patients did not answer phone calls or consent to the study raising questions about its generalisability, scalability and durability [36]. Of note, VICTORION-Spirit is an ongoing pragmatic, multicentre, randomised trial that will evaluate the implementation, patient experience and delivery of inclisiran (NCT04807400). The trial will also assess the impact of phone-based behavioural support, delivered by health advisors trained in motivational interviewing and coaching techniques. Motivational interviewing interventions in lipid management are currently heterogenous and their long-term durability requires further research [77].

### Simplifying Medication Regimens

Patients at high- or very high-risk of ASCVD are often treated with multiple lifelong medications and regimens can be complex [78]. There is increased interest in the polypill, a single pill containing two or more medication classes. In patients with ASCVD, a polypill intervention containing aspirin, ramipril and atorvastatin was associated with a significant reduction in ASCVD events compared with usual care, which may be due to greater medication adherence in the polypill group [79]. A retrospective analysis of administrative databases for a catchment of ~ 7 million people in Italy evaluated whether implementation of a lipid-lowering polypill improves adherence [37]. In 1,219 patients, the real-world study showed that switching to a single-pill combination of rosuvastatin and ezetimibe was associated with a significantly greater proportion of adherent patients compared with the free-pill combination over a 12-month period (75.2% vs 51.8%; *p* < 0.001) [37]. Simplifying medication regimens may be a valid strategy to improve lipid management [37, 80]. Simplifying medication regimens not only involves alleviating pill burden, but can also be accomplished by medication packaging (e.g., blister packs), greater duration of medication supply or by reducing the number of doses [81, 82]. Limited real-world data suggests that adherence to monoclonal antibodies targeting PCSK9 (e.g., evolocumab or alirocumab) appears to be better than statins, possibly contributed by their less frequent dosing [83]. Here, inclisiran may be more convenient to patients and theoretically improve adherence due to its six-monthly dosing regimen, but studies are required [84].

### Reminders, Behavioural Nudges and Feedback

Lipid guidelines recommend the utilisation of multiple interventions that promote adherence, including telephone, calendar and electronic device reminders [8, 9]. Interventions using mobile health technologies can be a scalable way to improve adherence to lipid-lowering therapies [85]. The impact of text messages to deliver education and improve medication adherence in patients with ASCVD is of interest, given that it is a simple and low-cost intervention requiring minimal staff support [38, 86, 87]. However, a recent pragmatic multicentre randomised trial of 1,424 patients in Australia, TEXTMEDS, demonstrated that implementation of a customised and personalised text message-based intervention had no effect on medication adherence and LDL-cholesterol levels at 12 months after acute coronary syndrome [38]. These findings highlight that other factors may strongly influence adherence. Frequent reminders may result in alert fatigue and lack of effect. Importantly, the technical success of implementing the program across 18 centres and 3 different time zones in Australia was demonstrated [38]. Nudging patient decision-making may also help to improve medication adherence by altering behaviour and can be implemented with minimal human input [39, 88]. An example is the ENCOURAGE trial, which randomised 182 patients attending cardiology clinics in the United States to personalised nudges via an electronic platform using artificial intelligence or usual care [39]. After 12 months, the proportion of days covered by statin therapy was significantly greater in the nudge group compared with usual care (0.74 vs 0.64; *p* = 0.04) [39]. In addition, daily alarms from electronic pill monitoring devices combined with patient or social partner feedback may be a low-cost and scalable intervention that improves adherence to statins in the short-term [40].

## System-level Strategies

### Electronic Health Record Alerts

Electronic health record alerts are widely utilised to facilitate patient care, and their data can be leveraged to identify patients that may require initiation or intensification of lipid-lowering therapies [89, 90]. Health systems can integrate automated alerts or nudges into existing technology to influence clinician decision-making; these can then be maintained with minimal resources [41]. A recent cluster randomised trial of 28 primary care practices including 4,131 patients in the United States found that nudge to clinicians (with and without a patient nudge) via electronic health records increased the initiation of statins, whilst nudge to patients alone was ineffective [41]. Yet, another cluster randomised trial where active and passive decision support interventions were implemented within an electronic health record did not change statin prescribing after 6 months among 82 cardiologists in the United States [42]. Also, the PROMPT-LIPID trial cluster-randomised 96 cardiovascular and internal medicine clinicians in the United States to receive electronic health record alerts with recommendations for lipid-lowering or no alerts for outpatients with ASCVD and LDL-cholesterol ≥ 70 mg/dL (1.8 mmol/L) (*n* = 2,500) [43]. The proportion of patients where lipid-lowering therapy was intensified at 90 days was numerically higher in the alert group compared with the no alert group, but this was not statistically significant (14.1% vs 10.1%; *p* = 0.08) [43]. Intensification was twofold greater among patients whose clinicians (*n* = 46) did not dismiss the alerts (21.2% vs 10.4%; *p* < 0.001), suggesting the need for strategies to reduce dismissal or alert fatigue [43, 91]. These trials form a basis for future implementation research in electronic records which is attractive for its low cost and potential scalability [42, 43].

### Financial Incentives

Financial incentives for patients may be a way of improving adherence but studies have shown mixed results [44]. Randomised trials have utilised insights from behavioural economic theory, management, and psychology to design financial incentives [44, 45]. A randomised trial in the United States showed that shared financial incentives for both patients and physicians modestly reduced LDL-cholesterol levels more than control and more than physician-only or patient-only incentives [44]. The success of shared financial incentives highlights the importance of strategies targeting both physician and patient factors. However, a more recent randomised trial of financial incentives lasting 6 months for 805 patients at increased ASCVD risk, suboptimal LDL-cholesterol levels and imperfect adherence was recently conducted in the United States [45]. After 6 months, adherence was greater in those receiving financial incentives (measured by opening of electronic pill bottles), but the change in LDL-cholesterol from baseline to 12 months (i.e., after ceasing financial incentives) was not significantly different compared with the control group [45]. The investigators recommended that future implementation research measure health outcomes rather than simply adherence [45]. Whilst the optimal strategy and population to target remains unclear for financial incentives, such a strategy may not be durable given its ongoing cost and complexity. Gamification as another form of incentive could be applicable to lipid management, but studies are required [92–94].

### Access to Healthcare and Therapies

Reduced access to healthcare can have significant implications for patients with chronic diseases. Telehealth is one of many methods of increasing access to healthcare [95]. Telehealth services for lipid management has expanded following the COVID-19 pandemic but well-conducted implementation research is needed to address barriers and facilitators to optimal telehealth interventions [96]. A recent prospective study showed that implementation of a virtual lipid clinic with intensive follow-up was associated with 95.4% and 81.5% of 346 patients with acute coronary syndrome attaining LDL-cholesterol < 70 mg/dL (1.8 mmol/L) and < 55 mg/dL (1.4 mmol/l), respectively [46]. Moreover, another recent study found that a remotely delivered hypertension and lipid management program in 10,803 patients in the United States resulted in significant reductions in blood pressure and LDL-cholesterol compared with education only [47]. The model of remote care delivery (navigator-led and pharmacist-supported) was scalable at a population level and met the needs of a diverse multi-ethnic cohort [47]. The patients incurred no extra costs except for copayments required by insurance for medications and tests [47]. However, patient engagement and retention remain a challenge for remote programs.

Furthermore, 1 in 8 patients with ASCVD report nonadherence, including skipping of doses, taking lower than prescribed doses, and delaying refilling of prescriptions due to costs [97]. Whilst the availability of PCSK9-directed therapies and the evidence supporting their use has increased, reimbursement restrictions and cost remain important barriers to their uptake [98]. Policy reforms, including changes in pricing regulations and reimbursement can enhance the affordability and streamline the regulatory process for lipid-lowering medications; this is particularly important for PCSK9-directed therapies [99–102]. Future research should evaluate whether innovative strategies that involve community pharmacies can help to improve access to PCSK9-directed therapies [103]. In addition, financial medication assistance may have a role in lipid management but implementation research is also required [104]. Another approach to achieving lower levels of LDL-cholesterol from a population health perspective is to allow statins to be over-the-counter. In the TACTiC trial, participant use of web app to establish safety and eligibility for a statin was concordant with blinded physician assessment and allowed access to rosuvastatin 5 mg daily without a prescription [48]. Participants subsequently reduced their LDL-cholesterol by ~ 35% and had high levels of adherence (> 95% days covered) owing to their high levels of baseline motivation [48]. While such an approach would need regulatory approval, studies among individuals with varying levels of health literacy will need to be performed to ensure such an approach is safe and equitable.

### Regular Monitoring of LDL-cholesterol

Regular lipid monitoring is often utilised to assess an individual’s response to lipid-lowering therapy in those who are adherent. Setting therapeutic goals through shared decision-making and arranging follow-up testing can facilitate behavioural change and improve medication adherence [105]. Indeed, measurement of LDL-cholesterol may be a high-value and low-cost intervention to improve adherence to statin therapy in patients with ASCVD [106, 107]. At the clinician level, LDL-cholesterol monitoring can also reduce therapeutic inertia related to the initiation and intensification of lipid-lowering therapies [106–108]. Implementation of a mobile device-based intervention including serial LDL-cholesterol levels, virtual follow-up and optimisation of therapy in a single centre in Spain was associated with > 70% of patients with acute coronary syndrome attaining LDL-cholesterol ≤ 70 mg/dL (1.8 mmol/L) [49]. Moreover, some health systems have transitioned to value-based care models that reward the achievement of quality outcomes at lower costs, thereby providing incentive to clinicians [109]. The re-establishment of national metrics to include LDL-cholesterol measurement and goal attainment as a necessary performance measure for patients and clinicians could therefore prove beneficial in improving population-wide lipid management [109].

## Future Directions

There is a need to incorporate implementation recommendations into lipid guidelines and consensus statements, which can be translated into meaningful implementation research [19, 110]. Guidelines will need to specify what care should be delivered and how best to implement its delivery [19]. Recently, models have been developed to assist guidelines in embedding implementation recommendations [19, 110]. Current implementation research is generally conducted in single centres limiting generalisability and often employs lower quality methodological study design such as pre-post comparison. Future implementation research in lipid management could benefit from more rigorous and innovative study designs, such as the stepped-wedge, counterbalanced, adaptive intervention or SMART (sequential multiple assignment randomised trials) designs for example [24, 110, 111]. Studies of interventions to improve lipid management should also evaluate longer-term outcomes and sustainability (e.g., > 1 year), given that most studies thus far have described relatively short follow-up. Future strategies for optimising lipid management could further utilise precision medicine (e.g., genetic and biomarker testing) and artificial intelligence (e.g., chatbots) approaches [112–114]. A greater focus should also be placed on multilevel and multifaceted interventions, which are generally superior to interventions that focus on single elements, given that behavioural change at multiple levels of the health system is required [115]. To inform implementation efforts, there is a need to understand how and why strategies are successfully implemented in some settings, but not others [116]. Increased efforts should therefore be placed in aligning future research with implementation science theories, models and/or frameworks [23, 110, 117–120]. Of note, only one study reviewed here (Jones, et al.) explicitly reported using an implementation framework (RE-AIM) [26]. By utilising an implementation science theory, model and/or framework, implementation studies can help researchers better understand the impact, sustainability and scalability of strategies, and where barriers and facilitators to lipid management may exist in other contexts [26]. Finally, harmonisation of international lipid guidelines has also been recommended as another potential solution [121].

## Conclusions

Lowering LDL-cholesterol is crucial for preventing ASCVD. However, many high- and very high-risk patients are not optimally managed according to guideline recommendations. Many barriers and facilitators to lipid management have been identified and implementation science can be leveraged to design and implement strategies to combat this. However, the feasibility and effectiveness of the implementation strategies reviewed have been variable. Multifaceted strategies with a systematic approach to targeting clinician, patient and system factors may be more successful. Several insights have been gained from studies of implementation strategies so far, but more work is still needed. Future research should continue to explore how to best adopt, integrate, maintain and refine implementation strategies that improve lipid management and therefore bridge the gap between evidence and clinical practice.

## Key References

- Hess CN, Daffron A, Nehler MR*, *et al.: Randomized Trial of a Vascular Care Team vs Education for Patients With Peripheral Artery Disease. J Am Coll Cardiol 2024, 83(25):2658–2670.
  - ○ Findings from this study demonstate the value of an interdisciplinary team approach to LDL-cholesterol lowering in patients with peripheral arterial disease.
- Jones LK, McMinn M, Kann D, et al.: Evaluation of a multidisciplinary lipid clinic to improve the care of individuals with severe lipid conditions: a RE-AIM framework analysis. Implement Sci Commun 2021, 2(1):32.
  - ○ Findings from this study, utilising a RE-AIM framework analysis, highlight the impact and likely sustainability of a multi-disciplinary lipid clinic.
- Blood AJ, Cannon CP, Gordon WJ, et al.: Results of a Remotely Delivered Hypertension and Lipid Program in More Than 10 000 Patients Across a Diverse Health Care Network. JAMA Cardiol 2023, 8(1):12–21.
  - ○ Findings from this study show the potential for a low-touch, scalable and remote intervention to achieve lipid goals.

## Data Availability

No datasets were generated or analysed during the current study.
